# Highly Frequent Mutations in Negative Regulators of Multiple Virulence Genes in Group A Streptococcal Toxic Shock Syndrome Isolates

**DOI:** 10.1371/journal.ppat.1000832

**Published:** 2010-04-01

**Authors:** Tadayoshi Ikebe, Manabu Ato, Takayuki Matsumura, Hideki Hasegawa, Tetsutaro Sata, Kazuo Kobayashi, Haruo Watanabe

**Affiliations:** 1 Department of Bacteriology I, National Institute of Infectious Diseases, Tokyo, Japan; 2 Department of Immunology, National Institute of Infectious Diseases, Tokyo, Japan; 3 Department of Pathology, National Institute of Infectious Diseases, Tokyo, Japan; Children's Hospital Boston, United States of America

## Abstract

Streptococcal toxic shock syndrome (STSS) is a severe invasive infection characterized by the sudden onset of shock and multiorgan failure; it has a high mortality rate. Although a number of studies have attempted to determine the crucial factors behind the onset of STSS, the responsible genes in group A *Streptococcus* have not been clarified. We previously reported that mutations of *csrS/csrR* genes, a two-component negative regulator system for multiple virulence genes of *Streptococcus pyogenes*, are found among the isolates from STSS patients. In the present study, mutations of another negative regulator, *rgg*, were also found in clinical isolates of STSS patients. The *rgg* mutants from STSS clinical isolates enhanced lethality and impaired various organs in the mouse models, similar to the *csrS* mutants, and precluded their being killed by human neutrophils, mainly due to an overproduction of SLO. When we assessed the mutation frequency of *csrS*, *csrR*, and *rgg* genes among *S. pyogenes* isolates from STSS (164 isolates) and non-invasive infections (59 isolates), 57.3% of the STSS isolates had mutations of one or more genes among three genes, while isolates from patients with non-invasive disease had significantly fewer mutations in these genes (1.7%). The results of the present study suggest that mutations in the negative regulators *csrS/csrR* and *rgg* of *S. pyogenes* are crucial factors in the pathogenesis of STSS, as they lead to the overproduction of multiple virulence factors.

## Author Summary

Group A *streptococcus* (GAS) causes life-threatening severe invasive diseases, including necrotizing fasciitis and streptococcal toxic shock-like syndrome. Although many studies have attempted to determine factors that are crucial for the onset of streptococcal toxic shock syndrome (STSS), bacterial factors responsible for it have not been clarified. By comparing genome sequences of clinical GAS isolates from STSS with those of non-invasive infections, we showed that mutations of negative regulator genes (*csrS*, *csrR*, *rgg*) were detected at a high frequency of more than 50% in STSS isolates, but at a low frequency of less than 2% in non-invasive isolates. These mutations of negative regulators were found in various *emm*-genotyped STSS isolates but not in a particular *emm* genotype. These mutants enhanced virulence in mouse models. Such results indicated that mutations of bacterial negative regulators are crucial for the pathogenesis of STSS due to the overproduction of multiple virulence factors under the de-repressed conditions.

## Introduction

*Streptococcus pyogenes* (group A *Streptococcus*; GAS) is one of the most common human pathogens. It causes a wide variety of infections, ranging from uncomplicated pharyngitis and skin infections to severe and even life-threatening manifestations, such as necrotizing fasciitis (NF) and bacteremia. Several streptococcal virulence factors, including pyrogenic exotoxins, streptokinase, and streptolysins, are reportedly involved in these diseases. Streptococcal toxic shock syndrome (STSS) is a severe invasive infection that has been recently characterized by the sudden onset of shock and multiorgan failure; it has a high mortality rate, ranging from 30% to 70% [1]. There is controversy as to whether the cause of STSS largely depends on host factors or bacterial factors. Although many studies have sought to determine the crucial factors behind the onset of STSS, the responsible GAS genes have not been clarified.

Recently, we and others have reported that mutations in the *csrS* (*covS*) gene—a sensor gene of a two-component regulatory system—were detected in a panel of clinical isolates from severe invasive streptococcal infections, but not in non-STSS isolates [2]–[4]. Mutations in the gene caused an increased expression of various virulence genes; the upregulation of streptolysin O (SLO) induced necrosis of neutrophils and prompted the escape of *csrS* mutated strains from being killed by neutrophils, resulting in increased virulence in lethality in the mouse model [2]. Complementation of the wild *csrS* gene into *csrS*-mutated STSS isolates dramatically decreased their virulence in lethality [2]. Similarly, *csrR* (*covR*) mutations were found in the clinical isolates of STSS patients [5]. Such results suggest that *csrS/csrR* mutations are closely associated with the onset of STSS.

However, several study groups that investigated the *csrS*/*csrR* gene sequence in each STSS isolate [3],[4],[6],[7] also report that there is no mutation in the *csrS/csrR* gene of STSS isolates [4]. These results raise questions as to how frequently STSS isolates have mutations in the *csrS/csrR* genes in a mass of isolates, and what mutations other than *csrS/csrR* genes may be responsible for the onset of STSS.

In this study, we sequenced the *csrS/csrR* genes of 164 GAS strains that have been isolated from STSS patients in Japan since 1992. Almost one-half of the STSS isolates had a mutation in the *csrS/csrR* genes. In addition, we found a mutation in the *rgg* (*ropB*) gene, instead of the *csrS/csrR* genes, in the clinical isolates of patients with STSS. Since the *rgg* gene negatively regulates various virulence genes in a manner similar to that of the *csrS* gene, a mutation of the *rgg* gene in STSS clinical isolates increased the expression of several virulence genes and virulence in lethality in the mouse model. Such mutations were detected at a high frequency in more than 50% of STSS isolates. These findings suggest that mutations in the negative regulators such as *csrS/csrR* and *rgg* of *S. pyogenes* bring about overproduction of a number of virulence factors, such as SLO, and play a crucial role in the onset of STSS.

## Results

### Mutation frequency of the *csrS/csrR* gene in STSS isolates

In our previous study, we reported that there were various types of mutations in the *csrS* gene of *emm49* clinical isolates from STSS patients [2] and in the *csrR* gene in *emm3* clinical isolates from STSS patients [5]. These findings strongly suggest that *csrS/csrR* mutations play important roles in the pathogenesis of STSS. To evaluate the frequency of these *csrS/csrR* mutations in isolates from clinical cases of STSS [8], we sequenced the *csrS* and *csrR* genes in STSS clinical isolates from sterile sites (164 isolates) and non-STSS clinical isolates from non-sterile sites (59 isolates). The diagnoses, sites of bacteria isolation, and characteristics of *S. pyogenes* isolates are described in Table 1. Of the 164 STSS clinical *S. pyogenes* isolates, 55 isolates (*csrS*, 46 isolates; *csrS* + *rgg*, 9 isolates) (33.5%) had mutations in the *csrS* gene, 19 isolates (*csrR*, 13 isolates; *csrR* + *rgg*, 6 isolates) (11.6%) had mutations in the *csrR* gene, and 2 isolates (1.2%) had mutations in both genes (Tables 1 and 2). The *csrS* and *csrR* genes of these isolates had deletions, point mutations, or insertions that created non-functional CsrS and CsrR products, as shown previously [2],[4],[5]. Therefore, 76 isolates (46.3%) had mutations in the *csrS* and/or *csrR* genes, while the remaining 88 STSS isolates (53.7%) had mutations in neither *csrS* nor *csrR* (Tables 1 and 2). On the other hand, non-STSS GAS isolates had a significantly lower number of mutations in the genes [*csrS* mutation, 1.69% (1/59); *csrR* mutation, 0% (0/59); total, 1.69% (1/59); *p* = 0.00000000062 by χ^2^ analysis]. Although *csrS/csrR* mutations were more common among STSS isolates examined than among non-STSS isolates, they were not present in all STSS isolates. This may suggest that mutations in other regulatory genes may also be found among STSS isolates.

**Table 1:** Clinical isolates used in this study.

Diagnosis	NIH No. Strain name	Site of bacterial isolation	*emm* type	*csrR*	*csrS*	*rgg*	Increased SLO, production	CsrS/CsrR and Rgg amino acid sequence alterations	Accession No.	Reference
STSS	NIH136	blood	1	mut	+	+	+	CsrR, Arg→Ser at aa 119	CsrR, AB517819	This study
	NIH447	blood	1	mut	+	+	+	CsrR, Asp→Glu at aa 53	CsrR, AB517877	This study
	NIH44	blood	1	+	mut	+	+	CsrS, delete Glu at aa 252, and Leu→Val at aa 253	CsrS, AB517809	This study
	NIH73	blood	1	+	mut	+	+	CsrS, 5 bp delete = stop at aa 407	CsrS, same as TK76	This study
	NIH83	blood	1	+	mut	+	+	CsrS, 5 bp delete = stop at aa 407	CsrS, same as TK76	This study
	NIH102	ascites	1	+	mut	+	+	CsrS, 1 bp delete = stop at aa 76	CsrS, AB517817	This study
	NIH205	soft tissue	1	+	mut	+	+	CsrS, Gln→Arg at aa 388	CsrS, AB517823	This study
	NIH202-2	blood	1	+	mut	+	+	CsrS, 1 bp delete = stop at aa 35	CsrS, same as NIH156-1	This study
	NIH213-3	blood	1	+	mut	+	+	CsrS, 1 bp delete = stop at aa 35	CsrS, same as NIH156-1	This study
	NIH220-1	blood	1	+	mut	+	+	CsrS, Gly→Val at aa 457	CsrS, AB517828	[33]
	NIH222	soft tissue	1	+	mut	+	+	CsrS, Ala→Ser at aa 206	CsrS, AB517829	This study
	NIH235	blood	1	+	mut	+	+	CsrS, 1 bp delete = stop at aa 35	CsrS, same as NIH156-1	This study
	NIH243-1	blood	1	+	mut	+	+	CsrS, Gly→Arg at aa 291	CsrS, AB517834	This study
	NIH253-1	blood	1	+	mut	+	+	CsrS, 1 bp delete = stop at aa 457	CsrS, AB517835	This study
	NIH286	blood	1	+	mut	+	+	CsrS, Ile→Tyr at aa 381 and His→Arg at aa 437	CsrS, AB517845	This study
	NIH314	rubor site	1	+	mut	+	+	CsrS, 11 bp insert = stop at aa 39	CsrS, same as NIH287-1	This study
	NIH397	blood	1	+	mut	+	+	CsrS, 1 bp delete = stop at aa 35	CsrS, same as NIH156-1	This study
	TK1097	soft tissue	1	+	+	mut	+	Rgg, Lys→Asn at aa 45	Rgg, AB517806	This study
	NIH60	blood	1	+	+	mut	+	Rgg, Ser→Pro at aa 103	Rgg, AB517813	This study
	NIH91	blood	1	+	+	mut	+	Rgg, Tyr→Phe at aa 271	Rgg, AB517816	This study
	NIH186	blood	1	+	+	mut	+	Rgg, point mutation = stop at aa 69	Rgg, AB517824	[34]
	NIH293	blood	1	+	+	mut	+	Rgg, Cys→Tyr at aa 249	Rgg, AB517848	This study
	NIH374-2	wound	1	+	+	mut	+	Rgg, 1 bp insert = stop at aa 139	Rgg, AB517861	This study
	NIH390	soft tissue	1	+	+	mut	+	Rgg, 6 bp insert = insert Apn and Ile between aa 139 and aa 140	Rgg, AB517865	This study
	NIH409	blood	1	+	+	mut	+	Rgg, Val→Ala at aa 148	Rgg, AB517870	This study
	NIH445	blood	1	+	+	mut	+	Rgg, Leu→Pro at aa 95	Rgg, AB517876	This study
	NIH75	blood	1	mut	mut	+	+	CsrR, Ala→Asp at aa 111; CsrS, Pro→Lys at aa 220	CsrR, AB517814; CsrS, AB517815	This study
	NIH381-1	wound	1	mut	+	mut	+	CsrR, Ala→Val at aa 96; Rgg, Leu→Pro at aa 109	CsrR, AB517863; Rgg, AB517864	This study
	NIH366	blood	1	+	mut	mut	+	CsrS, 1 bp delete = stop at aa 35; Rgg, delete from aa 129 to aa 247	CsrS, same as NIH156-1; Rgg, AB517857	This study
	NIH17	blood	1	+	+	+	-	WT sequence		[35]
	NIH68	blood	1	+	+	+	-	WT sequence		This study
	NIH94-2	blood	1	+	+	+	-	WT sequence		This study
	NIH111	blood	1	+	+	+	-	WT sequence		This study
	NIH135	soft tissue	1	+	+	+	-	WT sequence		This study
	NIH150	Joint fluid	1	+	+	+	-	WT sequence		This study
	NIH153	wound	1	+	+	+	-	WT sequence		This study
	NIH165-1	blood	1	+	+	+	-	WT sequence		This study
	NIH185	blood	1	+	+	+	-	WT sequence		This study
	NIH187	blood	1	+	+	+	-	WT sequence		[36]
	NIH188-1	blood	1	+	+	+	-	WT sequence		This study
	NIH195	amniotic fluid	1	+	+	+	-	WT sequence		This study
	NIH201	Joint fluid	1	+	+	+	-	WT sequence		This study
	NIH204-1	muscle	1	+	+	+	-	WT sequence		This study
	NIH214	blood	1	+	+	+	-	WT sequence		This study
	NIH223	soft tissue	1	+	+	+	-	WT sequence		This study
	NIH224	effusion	1	+	+	+	-	WT sequence		This study
	NIH225	blood	1	+	+	+	-	WT sequence		[33]
	NIH242	soft tissue	1	+	+	+	-	WT sequence		[36]
	NIH270	pleural effusion	1	+	+	+	-	WT sequence		This study
	NIH261	blood	1	+	+	+	-	WT sequence		This study
	NIH291-1	blood	1	+	+	+	-	WT sequence		This study
	NIH298	soft tissue	1	+	+	+	-	WT sequence		This study
	NIH304	blood	1	+	+	+	-	WT sequence		This study
	NIH315	fluid	1	+	+	+	-	WT sequence		This study
	NIH320	soft tissue	1	+	+	+	-	WT sequence		This study
	NIH324-2	blood	1	+	+	+	-	WT sequence		This study
	NIH342	blood	1	+	+	+	-	WT sequence		This study
	NIH338	blood	1	+	+	+	-	WT sequence		This study
	NIH344-1	blood	1	+	+	+	-	WT sequence		This study
	NIH354	fascia	1	+	+	+	-	WT sequence		This study
	NIH361	blood	1	+	+	+	-	WT sequence		This study
	NIH363	blood	1	+	+	+	-	WT sequence		This study
	NIH380-2	blood	1	+	+	+	-	WT sequence		This study
	NIH392	serum	1	+	+	+	-	WT sequence		This study
	NIH388-2	ascites	1	+	+	+	-	WT sequence		This study
	NIH395-1	blood	1	+	+	+	-	WT sequence		This study
	NIH399-1	pleural effusion	1	+	+	+	-	WT sequence		This study
	NIH413	soft tissue	1	+	+	+	-	WT sequence		This study
	NIH415	blood	1	+	+	+	-	WT sequence		This study
	NIH417-3	blood	1	+	+	+	-	WT sequence		This study
	NIH418	soft tissue	1	+	+	+	+	WT sequence		This study
	NIH436	soft tissue	1	+	+	+	-	WT sequence		This study
	NIH444	soft tissue	1	+	+	+	-	WT sequence		This study
	NIH9	blood	3	mut *	+	+	-	CsrR, Gln→Pro at aa 216	CsrR, AB219966	[35]
	NIH212	soft tissue	3	mut	+	+	+	CsrR, Asp→Tyr at aa 60	CsrR, AB517826	This study
	NIH216	fascia	3	mut	+	+	-	CsrR, Trp→Cys at aa 184	CsrR, AB517827	This study
	NIH259	blood	3	mut	+	+	-	CsrR, point mutation = stop at aa 45	CsrR, AB517839	This study
	NIH300	blood	3	mut	+	+	+	CsrR, Arg→Leu at aa 119	CsrR, AB517850	This study
	NIH404	soft tissue	3	mut	+	+	-	CsrR, 1 bp insert = stop at aa 146	CsrR, AB517867	This study
	TK280	blood	3	+	mut	+	+	CsrS, point mutation = stop at aa 131	CsrS, AB517803	This study
	NIH152-3	blood	3	+	mut	+	+	CsrS, point mutation = stop at aa 160	CsrS, AB517820	[37]
	NIH249	blood	3	+	mut	+	+	CsrS, 1 bp delete = stop at aa 35	CsrS, same as NIH156-1	This study
	NIH424-1	blood	3	+	mut	+	+	CsrS, 11 bp insert = stop at aa 39	CsrS, AB517873	This study
	NIH453	effusion	3	+	mut	+	+	CsrS, 1 bp delete = stop at aa 180	CsrS, AB517875	This study
	NIH3	blood	3	+	+	mut	+	Rgg, Tyr→Cys at aa 31	Rgg, AB517795	[35]
	NIH8	blood	3	+	+	mut	+	Rgg, Ile→Phe at aa 162	Rgg, AB517798	[35]
	TK3	soft tissue	3	+	+	mut	+	Rgg, Tyr→Cys at aa 31	Rgg, same as NIH3	This study
	TK64	fascia	3	+	+	mut	+	Rgg, Ile→Phe at aa 162	Rgg, same as NIH8	This study
	NIH34	blood	3	+	+	mut	+	Rgg, Ile→Phe at aa 162	Rgg, same as NIH8	[35]
	TK1153	blood	3	+	+	mut	+	Rgg, Ile→Phe at aa 162	Rgg, same as NIH8	This study
	NIH357	soft tissue	3	+	+	mut	+	Rgg, Phe→Tyr at aa 161	Rgg, AB517856	This study
	NIH1	fascia	3	mut	+	mut	+	CsrR, Arg→Cys at aa 118; Rgg, Tyr→Cys at aa 31	BA000034	[35]
	TK283	fascia	3	mut	+	mut	+	CsrR, point mutation = stop at aa 134; Rgg, Ile→Phe at aa 162	CsrR, AB517797; Rgg, same as NIH8	This study
	NIH18	effusion	3	+	mut	mut	+	CsrS, Ala→Tyr at aa 456; Rgg, Arg→Lys at aa 28	CsrS, AB517801; Rgg, AB517802	[35]
	NIH14	blood	3	+	+	+	-	WT sequence		[37]
	NIH16	muscle	3	+	+	+	-	WT sequence		[35]
	NIH21	blood	3	+	+	+	-	WT sequence		[35]
	NIH158	soft tissue	3	+	+	+	-	WT sequence		[37]
	NIH382-1	blood	3	+	+	+	-	WT sequence		This study
	NIH406	blood	4	mut	+	mut	+	CsrR, Asp→Gln at aa 53; Rgg, Val→Phe at aa 169	CsrR, AB517868; Rgg, AB517869	This study
	NIH307	wound	4	+	+	+	-	WT sequence		This study
	NIH432	Joint fluid	4	+	+	+	-	WT sequence		This study
	NIH296	blood	6	+	mut	+	+	CsrS, Met→Ile at aa 228 and Gly→Asp at aa 357	CsrS, AB517847	This study
	NIH323-1	lung	11	mut	+	+	+	CsrR, Asp→Gly at aa 10	CsrR, AB517853	This study
	NIH49	soft tissue	11	+	mut	+	+	CsrS, point mutation = stop at aa 184	CsrS, AB517810	This study
	NIH325-1	blood	11	+	mut	+	+	CsrS, point mutation = stop at aa 450	CsrS, AB517854	This study
	NIH50	blood	12	+	+	mut	-	Rgg, Glu→Asp at aa 89	Rgg, AB517811	This study
	NIH61	soft tissue	12	+	+	mut	-	Rgg, Glu→Asp at aa 89	Rgg, same as NIH50	[33]
	NIH109	Joint fluid	12	+	+	mut	-	Rgg, Glu→Asp at aa 89	Rgg, same as NIH50	This study
	NIH120	soft tissue	12	+	+	mut	-	Rgg, Glu→Asp at aa 89	Rgg, same as NIH50	This study
	NIH277	blood	12	+	+	mut	-	Rgg, Glu→Asp at aa 89	Rgg, same as NIH50	This study
	NIH383	blood	12	+	+	mut	-	Rgg, Glu→Asp at aa 89	Rgg, same as NIH50	This study
	NIH391	blood	12	+	+	mut	-	Rgg, Glu→Asp at aa 89	Rgg, same as NIH50	This study
	NIH398-2	blood	12	+	+	mut	-	Rgg, Glu→Asp at aa 89	Rgg, same as NIH50	This study
	NIH419	soft tissue	12	+	+	mut	-	Rgg, Glu→Asp at aa 89	Rgg, same as NIH50	This study
	NIH263-2	blood	12	+	mut	mut	+	CsrS, Asn→Lys at aa 384; Rgg, Glu→Asp at aa 89	CsrS, AB517840; Rgg, same as NIH50	This study
	NIH43	effusion	18	mut	+	mut	+	CsrR, Ser→Pro at aa 154; Rgg, Cys→Arg at aa 227	CsrR, AB517807; Rgg, AB517808	This study
	TK76	soft tissue	22	+	mut	+	+	CsrS, 5 bp delete = stop at aa 407	CsrS, AB517800	This study
	NIH160	blood	22	+	mut	+	+	CsrS, 1 bp delete = stop at aa 35	CsrS, same as NIH156-1	This study
	NIH172	blood	22	+	mut	+	+	CsrS, 1 bp delete = stop at aa 35	CsrS, same as NIH156-1	This study
	NIH403	blood	22	+	mut	+	+	CsrS, point mutation = stop at aa 369	CsrS, AB517866	This study
	NIH236	blood	22	+	mut	mut	+	CsrS, Change TTTTT to GAGG = stop at aa158; Rgg, Phe→Leu at aa 150	CsrS, AB517831; Rgg, AB517832	This study
	NIH98	blood	22	+	+	+	-	WT sequence		This study
	NIH429	blood	22	+	+	+	-	WT sequence		This study
	NIH35	blood	28	+	mut	+	+	CsrS, Glu→Gly at aa 226	CsrS, AB517805	[35]
	NIH40	blood	28	+	mut	+	+	CsrS, Glu→Gly at aa 226	CsrS, same as NIH35	This study
	NIH440	Joint fluid	28	+	mut	+	+	CsrS, Glu→Gly at aa 226	CsrS, same as NIH35	This study
	NIH422	soft tissue	28	+	mut	mut	+	CsrS, 1 bp delete = stop at aa 35; Rgg, Glu→Lys at aa 84	CsrS, same as NIH156-1; Rgg, AB517872	This study
	NIH423-1	blood	28	+	mut	mut	+	CsrS, 1 bp delete = stop at aa 35; Rgg, Glu→Lys at aa 84	CsrS, same as NIH156-1; Rgg, same as NIH422	This study
	NIH142-5	blood	28	+	+	+	-	WT sequence		This study
	NIH316	soft tissue	28	+	+	+	-	WT sequence		This study
	NIH200-4	blood	49	+	mut *	+	+	CsrS, Gly→Ser at aa 461	CsrS, AB517825	[34]
	NIH230	blood	49	+	mut *	+	+	CsrS, Change GTTCTTTTTT to TCTGCATTTTC = stop at aa 39	CsrS, AB517830	[34]
	NIH269	soft tissue	49	+	mut *	+	+	CsrS, 11 bp insert = stop at aa 39	CsrS, same as NIH250-2	[38]
	NIH346	blood	49	+	+	+	-	WT sequence		This study
	NIH410	soft tissue	49	+	+	+	-	WT sequence		This study
	NIH389	soft tissue	53	+	mut	+	+	CsrS, 11 bp insert = stop at aa 39	CsrS, same as NIH250-2	This study
	TK65	fascia	58	+	+	mut	+	Rgg, Cys→Phe at aa 85	Rgg, AB517799	This study
	NIH273	blood	58	mut	+	mut	+	CsrR, Gly→Ser at aa 95; Rgg, Tyr→Cys at aa 135	CsrR, AB517842; Rgg, AB517843	This study
	NIH301	blood	59	mut	+	+	+	CsrR, Ile→Phe at aa 30	CsrR, AB517851	This study
	NIH317	blood	60	+	mut	+	+	CsrS, point mutation = stop at aa 282	CsrS, AB517852	This study
	NIH297	soft tissue	77	+	mut	+	+	CsrS, Thr→Ile at aa 266	CsrS, AB517849	This study
	NIH258	soft tissue	78	+	+	+	-	WT sequence		This study
	TK929	blood	81	mut	+	+	+	CsrR, Arg→Ser at aa 118	CsrR, AB517804	This study
	NIH156-1	blood	81	+	mut	+	+	CsrS, 1 bp delete = stop at aa 35	CsrS, AB517821	This study
	NIH268	soft tissue	81	+	mut	+	+	CsrS, Arg→Cys at aa 241	CsrS, AB517841	This study
	NIH101	soft tissue	81	+	+	+	-	WT sequence		This study
	NIH283-1	blood	87	+	mut	+	+	CsrS, Pro→Leu at aa 16	CsrS, AB517844	This study
	NIH437	blood	87	+	mut	+	+	CsrS, Ser→Pro at aa 246	CsrS, AB517862	This study
	NIH371	blood	87	+	mut	mut	+	CsrS, 5 bp delete = stop at aa 407; Rgg, Glu→Tyr at aa 2 and Ile→Val at aa 3	CsrS, same as TK76; Rgg, AB517858	This study
	NIH372	blood	87	+	mut	mut	+	CsrS, point mutation = stop at aa 193; Rgg, Ala→Thr at aa 245	CsrS, AB517859; Rgg, AB517860	This study
	NIH157	blood	89	mut	+	+	+	CsrR, Asp→Tyr at aa 10	CsrR, AB517822	This study
	NIH5	blood	89	+	mut	+	+	CsrS, 5 bp insert = stop at aa 459	CsrS, AB517796	[35]
	NIH58	Joint fluid	89	+	mut	+	+	CsrS, Val→Ala at aa 423	CsrS, AB517812	This study
	NIH238	soft tissue	89	+	mut	+	+	CsrS, Ser→Arg at aa 204	CsrS, AB517833	This study
	NIH421	blood	89	+	mut	+	+	CsrS, Arg→Cys at aa 229	CsrS, AB517871	This study
	NIH118	blood	89	+	+	mut	+	Rgg, Asp→Tyr at aa 174	Rgg, AB517818	This study
	NIH345	wound	89	mut	mut	+	+	CsrR, Arg→Cys at aa 94; CsrS, 1 bp delete = stop at aa 35	CsrR, same as NIH252-2; CsrS, AB517855	This study
	NIH250-2	blood	89	+	mut	mut	+	CsrS, 11 bp insert = stop at aa 39; Rgg, Tyr→His at aa 135	CsrS, AB517836; Rgg, AB517837	This study
	NIH208	blood	89	+	+	+	-	WT sequence		This study
	NIH256	blood	89	+	+	+	-	WT sequence		This study
	NIH252-2	muscle	91	mut	+	+	+	CsrR, Arg→Cys at aa 94	CsrR, AB517838	This study
	NIH287-1	soft tissue	112	+	mut	+	+	CsrS, 11 bp insert = stop at aa 39	CsrS, AB517846	This study
	NIH433	blood	113	+	mut	+	+	CsrS, 3 bp delete = delete Asp at aa 470	CsrS, AB517874	This study
non-invasive isolates	K01	pharyngitis	1	+	+	+	-	WT sequence		This study
	K02	pharyngitis	1	+	+	+	-	WT sequence		This study
	K03	pharyngitis	1	+	+	+	-	WT sequence		This study
	K04	pharyngitis	1	+	+	+	-	WT sequence		This study
	K11	pharyngitis	1	+	+	+	-	WT sequence		This study
	K12	pharyngitis	1	+	+	+	-	WT sequence		This study
	K13	pharyngitis	1	+	+	+	-	WT sequence		This study
	K14	pharyngitis	1	+	+	+	-	WT sequence		This study
	S1393	pharyngitis	1	+	+	+	-	WT sequence		This study
	S2582	bronchitis	1	+	+	+	-	WT sequence		This study
	S2638	bronchitis	1	+	+	+	-	WT sequence		This study
	OS02	pharyngitis	1	+	+	+	-	WT sequence		This study
	OS06	pharyngitis	1	+	+	+	-	WT sequence		This study
	OS15	pharyngitis	1	+	+	+	-	WT sequence		This study
	OS17	pharyngitis	1	+	+	+	-	WT sequence		This study
	OT3	vaginitis	1	+	+	+	-	WT sequence		This study
	OT7	pharyngitis	1	+	+	+	-	WT sequence		This study
	OT8	pharyngitis	1	+	+	+	-	WT sequence		This study
	OT5	tonsillitis	1	+	+	+	-	WT sequence		This study
	OT10	pharyngitis	1	+	+	+	-	WT sequence		This study
	OT11	scarlet fever	1	+	+	+	-	WT sequence		This study
	S1	pharyngitis	1	+	+	+	-	WT sequence		This study
	S4	pharyngitis	1	+	+	+	-	WT sequence		This study
	S13	pharyngitis	1	+	+	+	-	WT sequence		This study
	S14	pharyngitis	1	+	+	+	-	WT sequence		This study
	S15	pharyngitis	1	+	+	+	-	WT sequence		This study
	S16	pharyngitis	1	+	+	+	-	WT sequence		This study
	S25	pharyngitis	1	+	+	+	-	WT sequence		This study
	Se235	pharyngitis	1	+	+	+	-	WT sequence		This study
	F482	pharyngitis	1	+	+	+	-	WT sequence		This study
	Se202	tonsillitis	3	+	mut	+	+	CsrS, Val→Leu at aa 25, Leu→His at aa 26 and Phe→Leu at aa 28	CsrS, AB517643	This study
	K22	pharyngitis	3	+	+	+	-	WT sequence		[35]
	K23	pharyngitis	3	+	+	+	-	WT sequence		[35]
	K24	pharyngitis	3	+	+	+	-	WT sequence		[35]
	K25	pharyngitis	3	+	+	+	-	WT sequence		[35]
	K31	pharyngitis	3	+	+	+	-	WT sequence		[35]
	K32	pharyngitis	3	+	+	+	-	WT sequence		[35]
	K33	pharyngitis	3	+	+	+	-	WT sequence		[35]
	K34	pharyngitis	3	+	+	+	-	WT sequence		[35]
	K35	pharyngitis	3	+	+	+	-	WT sequence		[35]
	OT22	tonsillitis	3	+	+	+	-	WT sequence		This study
	OS29	pharyngitis	3	+	+	+	-	WT sequence		This study
	OT24	tonsillitis	3	+	+	+	-	WT sequence		This study
	OT28	scarlet fever	3	+	+	+	-	WT sequence		This study
	F495	pharyngitis	3	+	+	+	-	WT sequence		This study
	Se230	pharyngitis	4	+	+	+	-	WT sequence		This study
	F2362	pharyngitis	4	+	+	+	-	WT sequence		This study
	Se242	pharyngitis	6	+	+	+	-	WT sequence		This study
	F2446	pharyngitis	11	+	+	+	-	WT sequence		This study
	Se157	pharyngitis	11	+	+	+	-	WT sequence		This study
	Se233	pharyngitis	12	+	+	mut	-	Rgg, Glu→Asp at aa 89	Rgg, same as NIH50	This study
	F2369	pharyngitis	12	+	+	mut	-	Rgg, Glu→Asp at aa 89	Rgg, same as NIH50	This study
	StNo.205	pharyngitis	22	+	+	+	-	WT sequence		This study
	Se172	pharyngitis	28	+	+	+	-	WT sequence		This study
	F2324	pharyngitis	28	+	+	+	-	WT sequence		This study
	1566	pus	49	+	+ *	+	-	WT sequence		[38]
	Kurume51	pus	49	+	+ *	+	-	WT sequence		[38]
	KH1651	pus	49	+	+ *	+	-	WT sequence		[38]
	S26	pharyngitis	58	+	+	+	-	WT sequence		This study

STSS, streptococcal toxic shock-like syndrome; mut, mutation; SLO, streptolysin O; aa, amino acid; Ala, alanine; Arg, arginine; Asn, asparagine; Asp, aspartic acid; Cys, cysteine; Gln, glutamine; Glu, glutamic acid; Gly, glysine; His, histidine; Ile, Isoleusine; Leu, leucine; Lys, lysine; Met, methionine; Phe, phenylalanine; Pro, proline; Ser, serine; Thr, threonine; Trp, tryptophan; Tyr, Tyrosine; Val, valine; WT, wild type.

+ in *csrS*, *csrR*,and *rgg*, the same sequence as the wild typed gene of SF370. + in SLO, enhanced production. – in SLO, the same amount as the wild strain of SF370. Accession No. deposited in DDBJ.

*: Each gene of these isolates was presented in previous publications [2],[5].

**Table 2:** Mutation frequency in the *csrS/csrR* and *rgg* genes.

		No. of strains with mutation(s) in gene(s)(%)								
Isolates from patients with:		*csrS*	*csrR*	*rgg*	*csrS+csrR*	*csrS*+*rgg*	*csrR*+*rgg*	*csrS*+*csrR*+*rgg*	none	Total
STSS	Total	46 (28.0)	13 (7.9)	27(16.5)	2 (1.2)	9 (5.5)	6 (3.7)	0 (0)	61 (37.2)	164 (100)
	SLO (+)	46	9^b^	18	2	9	6	0	1	91
	Non-functional mutation	46 (28.0) a	13 (7.9) b	18 (11.0) c	2 (1.2)	9 (5.5)	6 (3.7)	0 (0)	0 (0)	94 (57.3)
Non-invasive diseases	Total	1 (1.7)	0 (0)	2 (3.4)	0 (0)	0 (0)	0 (0)	0 (0)	56 (94.9)	59 (100)
	SLO (+)	1	0	0	0	0	0	0	0	1
	Non-functional mutation	1 (1.7) a	0 (0) b	0 (0) c	0 (0)	0 (0)	0 (0)	0 (0)	0 (0)	1 (1.7)

a *csrS* mutation affects significantly the expression of *slo* gene [18], and the mutant shows mucoid colony because the expression of the capsule synthesis operon is increased [4],[39]. The function of CsrS was determined by colony morphology and by increase of SLO production.

b *csrR* mutation does not affect significantly the expression of *slo* gene [18]. The cause of SLO increase in the 9 *csrR* mutants was described in the section of Discussion. The *csrR* mutant shows mucoid colony because the expression of the capsule synthesis operon is increased [4],[39]. The function of *csrR* was determined by colony morphology.

c *rgg* mutation affects significantly the production of *slo* gene [Figure 1, [16]] and SpeB [14], [16]–[17]. The function of Rgg was determined by the increase of SLO production and the decrease of SpeB production.

### *rgg* or *csrS* mutations in STSS clinical isolates

To identify novel bacterial factors that may contribute to the pathogenesis of STSS, we next investigated the expression pattern of virulence factors in *S. pyogenes* isolates. We determined the sequence of the *csrS/csrR* genes from a panel of *emm*-matched GAS isolated from STSS patients; NIH1 (also called SSI-1), NIH3, NIH8, NIH34, NIH152-3, NIH249, NIH327, and NIH352 were clinical isolates of the *emm3* genotype from STSS and C500, OT22, and K33 were *emm3* non-STSS isolates (Tables 1 and S1). A mutation in the *csrS* gene was found in NIH152-3, NIH249, NIH327, and NIH352 of the STSS isolates; however, the other STSS and non-STSS GAS isolates had mutations in neither the *csrS* nor the *csrR* gene (data not shown). To determine whether other *emm*3 STSS strains have possible mutations in their genomes, we used comparative genome sequencing (CGS) [9], a microarray hybridization-based method developed to search for single-nucleotide polymorphisms (SNPs) and insertion–deletion sites within a genome between *emm3* STSS and non-STSS isolates. We found several genes with SNPs in the NIH1 genome in comparison with that of non-invasive isolates K33. Three (*codY*, *csrR* and *rgg*) of them had non-synonymous amino acid change in NIH1 but not in K33 and C500 (Table S2). We further sequenced these 3 genes in other non-invasive isolate, OT22 and STSS isolates, NIH3, NIH8 and NIH34. A couple of genetic differences which affect amino acid sequence were detected between the STSS and non-STSS GAS isolates (Table 3). All four STSS isolates (NIH1, NIH3, NIH8, and NIH34) had some difference in SPs1742 (Rgg) but not in non-STSS isolates (C500, OT22, and K33) (Table 3). SPs1742 is identified as the *rgg* gene, a transcriptional regulator of multiple genes [10]–[13], although the role of the *rgg* gene is controversial [14].

**Table 3:** Amino acid difference in comparison with intact ORF of SF370.

Isolates	Strain name	SPs0322 (CodY)	SPs1615 (CsrR)	SPs1742 (Rgg)	CsrS
Non-STSS isolates	C500	+	+	+	+
	OT22	+	+	+	+
	K33	+	+	+	+
STSS isolates	NIH1	-	-	-	+
	NIH3	-	+	-	+
	NIH8	+	+	-	+
	NIH34	+	+	-	+

(+): the same as the intact ORF of SF370 (accession No. AE004092)).

(-): difference from the intact ORF.

### Increased SLO expression in STSS isolates with *csrS* or *rgg* mutations

We [2] and others [4] have previously reported that STSS *emm49* and *emm1* clinical isolates exhibit a higher expression of the SLO gene (*slo*) than non-STSS isolates, due to a mutation in the *csrS* gene. Therefore, we examined whether a panel of *emm3*-genotyped STSS isolates possessing mutations in the *csrS* or *rgg* gene and *emm3* non-STSS isolates lacking mutations could produce SLO (i.e., secretion of SLO in the culture supernatant). The comparison of the supernatants at the same growth condition (data not shown) showed that larger amounts of SLO were secreted by STSS isolates possessing mutations in the *csrS* gene (NIH152, NIH249, NIH327, and NIH352) or *rgg* gene (NIH1, NIH3, NIH8, and NIH34) than by non-STSS isolates (C500, OT22, and K33) (Figure 1). These data suggest that *rgg* mutations may be related to an increased expression of SLO, as observed in the case of *csrS* mutations. To clarify the role of *rgg* gene mutation in STSS isolates in terms of SLO production, we created the *rgg* mutants K33*rgg* and OT22*rgg*, non-STSS isolates into which an *rgg* mutation had been introduced. They exhibited increased SLO secretion, as observe with STSS isolates (Figure 1). In contrast, when an intact *rgg* gene was integrated into the genomic DNA of the STSS isolates NIH8 and NIH34 (NIH8*rgg* ^+^ and NIH34*rgg* ^+^), the SLO secretion was decreased to the level of that in non-STSS isolates (Figure 1). Taken together, it appears that the mutation of the *rgg* gene was responsible for increased SLO production in the culture supernatant as that of *csrS* gene was.

**Figure 1:**
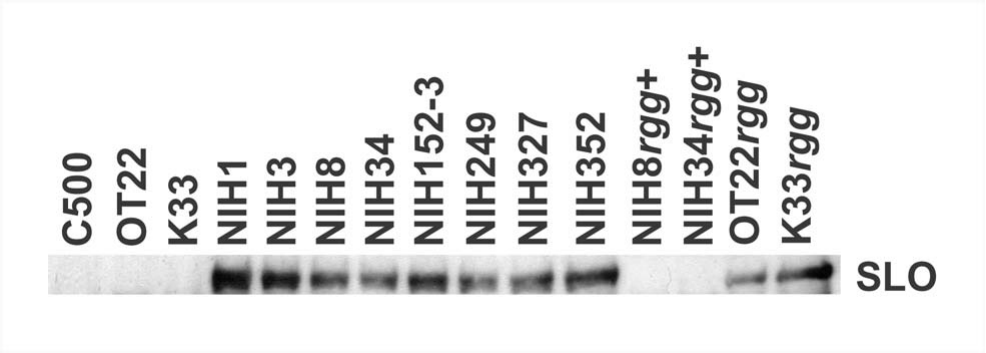
More SLO is secreted in STSS isolates than in isolates from non-invasive infections. The supernatants from an overnight culture (OD_600_ = 1.0) of *emm*3 *S. pyogenes* clinical isolates (non-STSS: C500, OT22, and K33; STSS: NIH1, NIH3, NIH8, NIH34, NIH152, NIH249, NIH327, and NIH352; non-STSS isolates with the mutated *rgg* gene: OT22*rgg* and K33*rgg*; STSS isolates complemented with the intact *rgg* gene: NIH8rgg^+^ and NIH34rgg^+^) were concentrated with trichloroacetic acid, and 5 µl of each sample was analyzed by western blotting with rabbit anti-SLO polyclonal antibody. Representative data of two independent experiments are shown.

### Enhanced expression of various virulence genes in STSS isolates is attributed to mutation of the *rgg* gene

**Figure 2:**
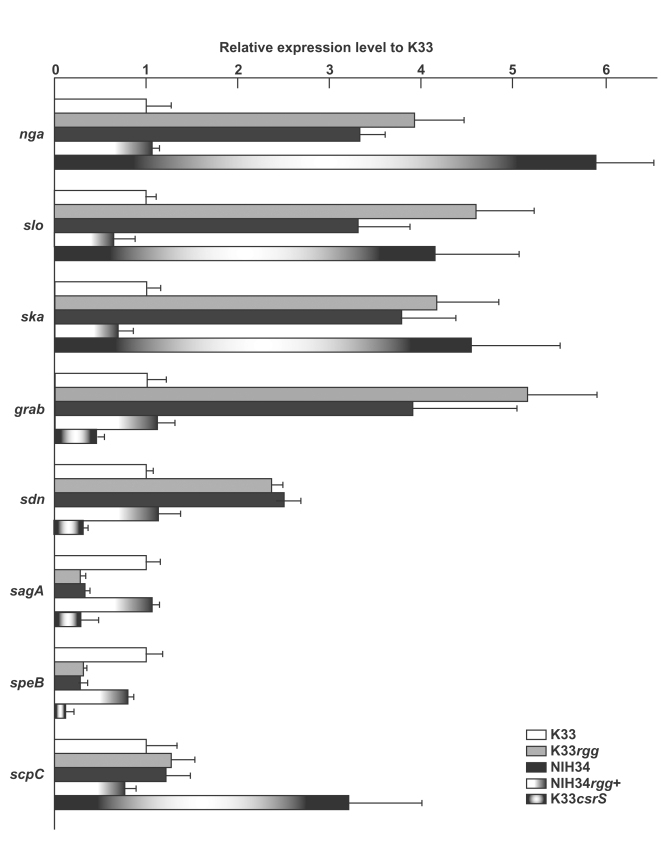
Mutation of the *rgg* gene influences expression of virulence-associated genes. The expression of virulence-associated genes in non-STSS, STSS GAS isolates, and strains into which an intact gene or mutant *rgg* or mutant *csrS* gene had been introduced was analyzed by RT-PCR; columns represent the relative mRNA expression levels of virulence-associated genes of each strain: nicotine adenine dinucleotide glycohydrolase (*nga*), streptolysin O (*slo*), streptokinase (*ska*), protein G-related alpha2-macroglobulin-binding protein (*grab*), streptodornase (phage-associated) (*sdn*), streptolysin S (*sagA*), streptococcal pyrogenic endotoxin (*speB*), and IL-8 protease (*scpC*). The expression level of K33 strain is shown as 1. Values are means ± SD (n = 4).

It has been reported that Rgg influences the transcription of many virulence-associated genes in *S. pyogenes* [10]–[13]. To test the possibility that the transcriptional expression levels of virulence genes are regulated by the function of the *rgg* gene, we performed real-time polymerase chain reaction (RT-PCR) with specific primers for each virulence-associated gene. The amounts of mRNA of protein G-related alpha2-macroglobulin-binding protein (*grab*), nicotine adenine dinucleotide glycohydrolase (*nga*), streptodornase (phage-associated) (*sdn*), streptokinase (*ska*), and *slo* in the STSS isolate of NIH34 with the *rgg* mutation were larger than those of the pharyngitis isolate of K33 with the intact *rgg* gene (Figure 2). On the other hand, the amounts of mRNA of the cystein protease (*speB*) and streptolysin S (*sagA*) genes in the STSS isolate of NIH34 were less than a half of those in the non-STSS isolate of K33 (Figure 2). The amounts of mRNA of the IgG-degrading protease of GAS, Mac-1-like protein (*mac*), C5a peptidase (*scpA*), IL-8 protease (*scpC*), superantigen (*speA*), and DNA gyrase (*gyrA*) genes in NIH34 were almost the same as those in K33 (Figure 2 and data not shown). NIH34*rgg* ^+^ suppressed the expression of virulence-associated genes to the levels found in non-STSS isolates; further, the expression of *speB* and *sagA* genes was increased to levels observed in non-STSS isolates (Figure 2). Additionally, the expression pattern of the virulence genes in K33*rgg* was similar to that in the STSS isolate NIH34 (Figure 2). These findings suggest that the transcriptional expression of multiple virulence genes, including the *slo* gene in GAS, was strongly influenced by the mutation in the *rgg* gene.

### *rgg* mutation is important in the pathogenesis of invasive infections in mouse models

**Figure 3:**
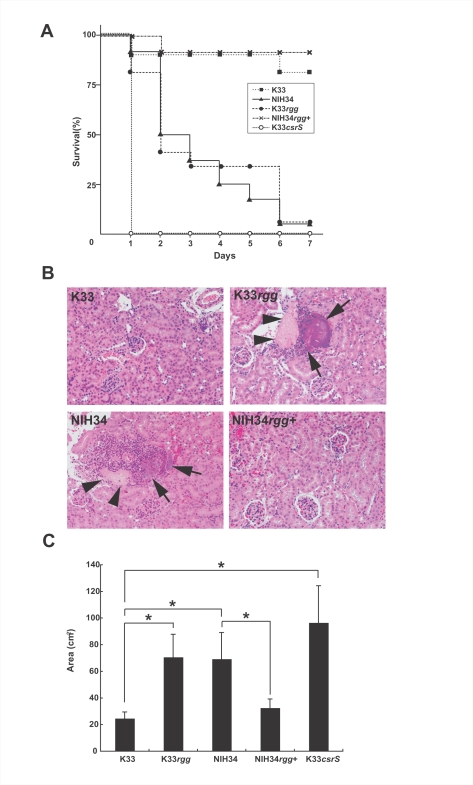
Mutation of *rgg* gene enhances the lethality and histopathology of GAS in mouse *in vivo* infection models. (A) Survival curves of mice infected with each strain. Mice were intraperitoneally inoculated with 1×10^7^ CFU of each GAS, and mouse survival was observed for 7 days post-infection. Mortality differences were statistically significant (P<0.01), as determined by a log-rank test. Survival curves were generated from 3 independent experiments using a total of 10–16 ddY mice for each strain. (B) Histopathological changes in the kidneys of mice infected with GAS. Tissue was extracted at 24 h after the intraperitoneal injection of GAS (1×10^7^ CFU). The black arrows indicate clusters of bacteria with filtrated inflammatory cells. The triangle heads indicate fibrous debris. (C) Lesion areas of subcutaneous infection in hairless mice injected with GAS. 1×10^7^ CFU in 100 µL suspension of GAS in PBS was injected subcutaneously, and the lesion area and body weight were measured each day after infection. The peak values are shown as means ± SD (n = 5). *The skin-lesion area in *rgg* mutant strains-infected mice was significantly larger than that in *rgg* intact strains (p<0.05), as estimated by ANOVA.

To elucidate the role of *rgg* in infections *in vivo*, we used GAS intraperitoneal injections to compare the lethality and histopathology of NIH34 with that of the K33 strain in a mouse model. The NIH34 strain showed significantly higher lethality than the K33 strain (*p* = 0.00027) (Figure 3A). Introduction of the *rgg* mutation in the K33 strain (K33*rgg*) resulted in higher lethality among infected mice than the K33 strain (*p* = 0.00067) and exerted a level of lethality similar to NIH34. The NIH34 strain into which an intact *rgg* gene (NIH34*rgg^+^*) had been introduced exhibited less lethality than the NIH34 strain (*p* = 0.0000097) and possessed the same level of lethality as the K33 strain. We confirmed that bacteria isolated from kidney or liver of infected mice at day 6 retained the mutation (data not shown). Therefore, the mutation of the *rgg* gene in the STSS isolates contributed to enhanced lethality in the mouse model. Histopathological examination of mice infected with NIH34 or K33*rgg* strains was carried out. Scattered multiple inflammatory foci containing bacterial colonies were observed in the kidney. The foci were accompanied with neutrophil infiltration, cell debris and hyalinization (Figure 3B). In contrast, no significant pathological change was observed in mice inoculated with the K33 or NIH34*rgg* ^+^ strains (Figure 3B). In another mouse model of soft-tissue infections, subcutaneous infection with NIH34 or K33*rgg* resulted in significantly larger lesions as compared to the infection with NIH34*rgg* ^+^ or K33 (*p*<0.01) (Figure 3C). Bacteria were isolated from spleen and kidney after the subcutaneous infection of the *rgg* mutants but not the intact *rgg* strains. We confirmed that bacteria isolated from lesions retained the mutation (data not shown).This showed that subcutaneous inoculation of mice led to the systemic spreading in the *rgg* mutant. These results suggest that the *rgg*-mutated strains isolated from STSS patients are more virulent *in vivo* than strains from patients with non-invasive infections, and that the increase in virulence *in vivo* is canceled by introducing an intact *rgg* gene.

### Mechanism of the resistance of *rgg* mutants to killing by human neutrophils

**Figure 4:**
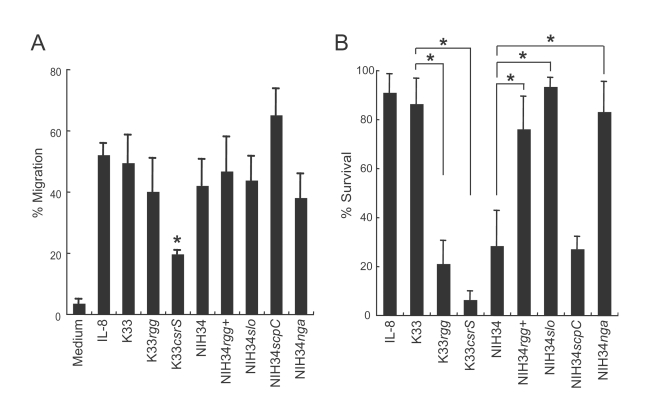
Effect of *rgg* and other mutations on migration and survival of human neutrophils. (A) The effect of human neutrophil migration in response to IL-8 by various GAS strains (K33, NIH34, and their *rgg*, *slo*, *scpC*, *nga*, and *csrS* mutants) was analyzed using a Transwell system and flow cytometry. About 62% of applied human neutrophils migrated through the Transwell, under the conditions of IL-8 addition. Values shown are means ± SD (n = 3). *p<0.05, as estimated by ANOVA. The results shown are representative of one of five individual experiments, all of which had similar results. (B) The viability of human neutrophils in the lower wells of a Transwell system, after migration in response to IL-8. The migrated human neutrophils were bought into contact with various GAS strains (K33, NIH34, and their *rgg*, *slo*, *scpC*, *nga*, and *csrS* mutants), and the remaining viable neutrophils were counted. Values shown are means ± SD (n = 3). *p<0.05, as estimated by ANOVA.

In our previous study, using the Transwell system, we showed that SLO, which causes necrosis in neutrophils, and an IL-8 protease of ScpC are important for bacterial resistance to killing by neutrophils [2]. Here, we examined the effect of *rgg* mutation on resistance to killing by neutrophils. As shown in Figure 4A, the migration ability of human neutrophils in response to chemokine IL-8 did not significantly differ between K33 and K33*rgg* or between NIH34 and NIH34*rgg* ^+^. Furthermore, the *scpC* mutation in the NIH34 strain did not have a significant influence on the migration of human neutrophils, compared to the *csrS* mutation, as previously reported (Figure 4A). This finding is in accordance with the less influence of ScpC expression in the *rgg* mutation (Figure 2). Collectively, the mutation of the *rgg* gene had little influence on the migration of human neutrophils in response to IL-8. As previously reported [2], migrated neutrophils may be killed by the STSS GAS isolates via enhanced SLO production, and therefore we examined this possibility. Human neutrophils were efficiently killed by the *rgg*-mutated strains (NIH34 and K33*rgg*), whereas strains with the intact *rgg* gene (K33 and NIH34*rgg* ^+^) did not cause obvious impairment of neutrophils (Figure 4B). In the *slo*-deficient mutant, the ability to kill neutrophils was abolished. Nicotine adenine dinucleotide glycohydrolase (Nga) is a cytotoxic protein secreted through the SLO complex [15]. Based on the results that the *nga* expression was negatively regulated by the *rgg* gene (Figure 2), we examined the lethal activity of the *nga* mutant against neutrophils. The neutrophil-killing activity was significantly decreased in an *nga*-deficient mutant (NIH34*nga*), but to a lesser extent as compared to the activity of NIH34*slo*. Therefore, these findings strongly suggest that SLO is a factor essential for neutrophil-killing activity in *rgg*-mutated *emm*3 STSS isolates, and that Nga partially influences the neutrophil-killing activity.

### Comparison of virulence between the *csrS* and *rgg* mutations

In our previous study, a *csrS* mutation in the *emm49*-genotyped strains was a key to the onset of severe invasive streptococcal infections [2]. The *csrS* mutant showed higher lethality in a mouse model and more efficiently killed human neutrophils than the non-mutated strain [2]. Therefore, we next compared the effect of the mutation in the *csrS* gene with that in the *rgg* gene, in terms of *in vivo* virulence in lethality and impairment of neutrophil function *in vitro*. Intraperitoneal infection of mice with the *csrS* mutant (K33*csrS*) caused earlier death and higher lethality than did infection with the *rgg* mutant (K33*rgg*) (*p* = 0.017) (Figure 3A). Furthermore, K33*csrS* strains decreased the migration ability of neutrophils in response to IL-8, and they induced necrosis of migrated neutrophils to a greater degree than did the *rgg* mutants (Figures 4A, B). These and the aforementioned results suggest that the *rgg* mutant can escape being killed by neutrophils only because of the SLO function, and not because of ScpC, whereas both SLO and ScpC in the *csrS* mutant contribute to the escape. This suggests that the *csrS* mutant may be more virulent in systemic infections than the *rgg* mutant, owing to its ability to up-regulate more virulence factors such as ScpC (Figures 2 and 3A,B).

### Mutation frequency of the *rgg* and the *csrS/csrR* genes in STSS clinical isolates

In this study, we found that there are mutations in the *rgg* gene or the *csrS/csrR* genes in STSS clinical isolates. We sequenced the *rgg* gene in strains isolated from sterile sites of STSS patients and found that 42 of 164 (25.6%) isolates carried some mutations (deletion, point mutation, or insertion) in the *rgg* gene. To determine whether these mutations contributed to a loss of Rgg function, we examined the level of SLO and SpeB [14] secretion and compared it with that in non-STSS isolates because overproduction of SLO [This study, 16] and less secretion of SpeB are also reported in the *rgg* mutation [14], [16]–[17]. We defined these phenotyped isolates as Rgg non-functional mutants. In 33 of 42 isolates, SLO production had increased and SpeB production had decreased (Tables 1 and 2 and data not shown). All of remaining nine *rgg* mutants (strains with mutation only in *rgg*) showing no increase of SLO expression were *emm12*-genotyped strains and had a mutation at the same position in comparison with other non-invasive strains. This mutation was synonymous in the level of amino acid, so we defined the mutants are functional as shown in Table 2. Collectively, 11.0%, 28.0%, 7.9%, 1.2%, 5.5%, and 3.7% of the 164 STSS clinical isolates carried non-functional mutations in the *rgg*, *csrS*, *csrR*, both *csrS* and *csrR*, both *csrS* and *rgg*, and both *csrR* and *rgg* genes, respectively, so that a total of 57.3% of the STSS isolates carried mutations in one or more of these negative regulator genes (Tables 1 and 2). On the other hand, the frequency of mutations in these genes was very low (1.7%) in non-invasive isolates (Tables 1 and 2). Therefore, the incidence of mutations in these genes is higher in STSS isolates than in non-invasive isolates (*p*<0.01 by χ^2^ analysis). This finding suggests that mutations in the *csrS/csrR* genes or the *rgg* gene are crucial factors causing severe invasive infections, such as STSS.

## Discussion

Since the late 1980s, STSS caused by *S. pyogenes* has become a serious health problem in both developed and developing countries. In this study, we found a high frequency of mutations of negative regulators in STSS clinical isolates. The *rgg* mutant killed human neutrophils, impaired multiple organs, and enhanced lethality in the mouse model, similar to the *csrS* mutant. These findings suggest that the impairment of negative regulators of *S. pyogenes* virulence genes induces neutrophil incompetence and subsequent STSS infection. This study is the first to show that clinical *S. pyogenes* isolates from STSS patients have mutations in one or more of genes––*rgg*, *csrS*, and *csrR––*which are involved in the negative regulation of multiple virulence genes.

In our previous study, we found mutations in the *csrS*/*csrR* genes of 5 *emm49* strains isolated from patients with severe invasive infections [2]. In the present study, we further examined whether STSS isolates other than the *emm49* genotype possess mutations in the *csrS* and *csrR* genes: 46.3% of the STSS isolates including various *emm* genotypes had non-functional mutations in one or more of the *csrS/csrR* genes. This finding suggests that mutations in the *csrS/csrR* genes are commonly recognized in STSS clinical isolates with various *emm* genotypes.

We have shown that the amount of SLO protein produced in STSS isolates is greater than that in non-STSS isolates, and that this effect is due to mutations in both the *rgg* and *csrS* genes of the isolates. The loss of function incurred by the mutation in the *rgg* gene in *emm3*-genotyped *S. pyogenes* affected the regulatory network of the virulence-associated genes; hence, the mutated strains could resist killing by neutrophils and caused damage to various organs in the mouse models. Therefore, the mutated *emm3*-genotyped *S. pyogenes* strains may potentially cause severe infections such as STSS in humans. Hollands et al. [14] reported that a mutation of the *rgg* (*ropB*) gene reduces M1T1 group A streptococcal virulence. We examined the contribution of Rgg to the pathogenesis of systemic infections by using a clinical *emm1*-genotyped STSS isolate, NIH186, and an *emm1*-genotyped pharyngitis isolate, Se235. NIH186 and Se235*rgg*, both of which had a mutation in the *rgg* gene, showed higher lethality than NIH186*rgg* ^+^ and Se235, in both of which the *rgg* gene is intact (data not shown). The *rgg* mutants impaired neutrophils to a greater extent than the *rgg*-intact strains did (Figure S1); this finding suggests that *rgg* mutants are more virulent than *rgg*-intact strains, in the *emm1* genotype. Therefore, the discrepancy between the finding in this study and that of Hollands et al. [14] may be attributed to modified regulation of SLO expression in *rgg*-mutated isolate in the latter, but not downregulation of *speB* and *sagA* operons.

Rgg is reported to regulate the transcription of many virulence-associated genes in *S. pyogenes* [10]–[13], and its regulatory profile varies among strains used [16]–[17]. Nevertheless, up-regulation of the *slo*, *nga* and *ska* genes and down-regulation of the *speB* gene are commonly found in the *rgg* mutation of *emm3*-genotyped isolates (Figure 2) and of M49 serotyped-strains, NZ131 and CS101 [16]–[17], suggesting they are the Rgg core regulon of GAS strains.

**Figure 5:**
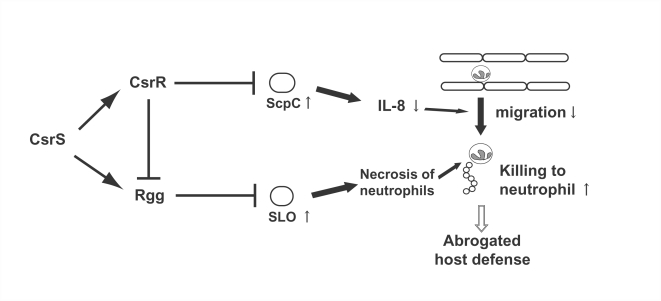
Schema of regulatory network and its dysfunction in STSS isolates leading to host evasion. CsrS phosphorylates CsrR, and the CsrR represses expression of a number of virulence genes including *rgg* and *scpC* [18]. CsrS also positively regulates the expression of *rgg* [4], which suppresses *slo* gene expression (Figure 2). The *rgg* mutation causes an overexpression of SLO, which kills neutrophils, but has no influence on ScpC expression. In the *csrR* mutant, overproduced ScpC inhibits the migration of neutrophils, and upregulated Rgg reduces the *slo* gene expression. In the *csrS* mutant, inactive form of CsrR leads to the overproduction of ScpC, which inhibits the migration of neutrophils, and decrease of Rgg leads to the overproduction of SLO, which kills neutrophils.

In recent studies, it has been reported that expression of the *rgg* gene is positively regulated by CsrS [4], while it is negatively regulated by CsrR [16]. Expression of the *slo* gene is enhanced in the *csrS* mutant (Figure 2) [2],[4], but not in the *csrR* mutant [18]. In this study, the expression of the *slo* gene was enhanced in the *rgg* mutant (Figure 2), suggesting that the enhancement of the *slo* gene may serve as the same regulatory pathway as the effect of the *csrS* mutation. These findings suggest that CsrS affects the Rgg regulon as well as the CsrR regulon (Figure 5); in the *csrS* mutant, CsrR is not phosphorylated by CsrS, and Rgg expression is suppressed.

It has been reported that the *csrR* null-mutation does not affect the expression of SLO [18]. However, Treviño et al. [19] reported that SLO production increases as a result of a *csrR* mutation in which histidine replaces arginine at position 119 of the CsrR protein; however the protein retained DNA-binding activity. The strains carrying such a kind of mutation are phenotypically identical to the *csrS* mutants [19]. Nine *csrR* mutants in this study showed increased SLO production (Tables 1 and 2), 2 (NIH136 and NIH300) of which had an amino acid replacement at position 119 of CsrR protein. Other 7 isolates showed mutation in the N-terminal amino acid of CsrR, but the exact mechanism of the CsrR mutant remains to be solved.

The *csrS*/*csrR* and *rgg* genes negatively regulate various virulence genes; however, they regulate different virulence genes. The *slo*, *nga*, and *ska* genes are negatively regulated by both CsrS/R and Rgg. The *grab* gene is negatively regulated by Rgg, while the *mac*, *scpA*, and *scpC* genes are negatively regulated by CsrS [2] (Figure 2). Thus, in terms of impairing neutrophil function, the *csrS* mutant inhibits the migration of neutrophils due to the destruction of IL-8 by the increased expression of *scpC* (Figure 5) [2]; meanwhile, the *rgg* mutant does not significantly affect the expression of *scpC*. On the other hand, since both *rgg* and *csrS* genes negatively regulate the expression of *slo*, infections with these mutants result in damage of neutrophils due to the increased production of SLO in the foci. This may explain why neutrophils are observed histopathologically in some cases of severe invasive infection, but are not in others. Indeed, our mouse model shows that neutrophils clustered around the foci of bacteria in the kidney infected by the *rgg* mutant (Figure 3B) but not by the *csrS* mutant [2].

The *slo*, *nga*, and *ska* genes are negatively regulated by both CsrS and Rgg [2] (Figure 2). We previously reported that SLO is an important virulence factor for the necrosis of neutrophils, which leads to higher lethality of infected mice [2]. Nucleosidase (NADase), which is encoded by the *nga* gene, contributes to severe invasive infections by GAS in the murine model of infection [20]. Streptokinase, which is encoded by the *ska* gene, has an important role in GAS invasion and proliferation [21]. STSS isolates carrying mutations in the *csrS* gene and/or the *rgg* gene commonly increased the expression of these genes [2; this study]. Thus, overproduction of these factors in the mutants could cooperatively contribute to increased virulence, thus causing the onset of STSS.

Notably, the mutation frequency of these genes in STSS isolates (57.3%) was much higher than that in non-invasive isolates (1.7%). These results suggest that mutations in the negative regulators of various virulence genes are important to the STSS onset. However, 42.7% of the STSS isolates did not have mutations in the *csrS/csrR* or *rgg* genes. Such strains may have mutations in other various other two-component regulatory systems or regulators in the *S. pyogenes* genome [22], which would be the focus of our research. We could not exclude the possibility that clinical severity of infection by strains lacking any mutations in the three genes depends on host factors, and not on bacterial factors. Specific human leukocyte antigen class II haplotypes are associated with a risk of disease severity [23], and the importance of both host and environmental factors has been reported [24].

In the mouse model, the *csrS* mutant (K33*csrS*) showed higher lethality than the *rgg* mutant. However, in the present study, the mortality rate of STSS patients infected with the *rgg* mutant was 60.9%, while that of patients infected with the *csrS* mutant was 47.2% (data not shown). These findings suggest that the *rgg* mutant also causes high lethality in humans, which may indicate differences in disease severity between humans and mice. Streptokinase is highly specific for human plasminogen, exhibiting little or no activity to those of other animal species [25]. Human-specific pathogenic factor(s) may influence virulence in cases of infection with the *rgg* mutant.

Collectively, we showed that mutations of negative regulators that result in the overproduction of multiple virulence factors are important to the onset of severe invasive infections such as STSS. Recently, it has been reported that community-associated methicillin-resistant *Staphylococcus aureus* (CA-MRSA) causes severe invasive infections, resulting in NF or even death [26],[27]. The enhanced virulence of CA-MRSA has been linked to an overproduction of leukolytic peptides, phenol-soluble modulins (PSMs) [28],[29]. The production of PSMs is regulated under the strict control of *agr* [29]. The change of expression of the *agr* regulator results in increased expression of virulence factors and increased virulence. Severe invasive infections are caused not only by *S. pyogenes* but also by other bacteria such as other *Streptococcus*, *Staphylococcus aureus*, *Vibrio vulnificus*, and *Aeromonas* spp. Such severe invasive infections may be caused by the coordinated overexpression of multiple virulence factors that are affected by the global regulatory network.

## Methods

### Ethic statement

This study complies with the guidelines of the declaration of Helsinki. This study protocol was approved by the institutional individual ethics committees for the use of human subjects (the National Institute of Infectious Diseases Ethic Review Board for Human Subjects) and the animal experiments (the National Institute of Infectious Diseases Animal Experiments Committee). Written informed consent was obtained from all study participants or their legal guardians for the patients who died. All clinical samples and healthy human neutrophils were stripped of personal identifiers not necessary for this study. All animal experiments were performed according to the Guide for animal experiments performed at National Institute of Infectious Diseases, Japan.

### Bacterial strains and culture conditions

The *S. pyogenes* strains and plasmids used in this study are described in Tables 1 and S1. The STSS criteria in this study are based on those proposed by the Working Group on Severe Streptococcal Infections [8]. The clinical isolates used were isolated from sterile sites of patients with STSS (164 isolates; age 0–99 years) and from non-sterile sites of patients with non-invasive infections (59 isolates; ages 1–67 years). The isolates from STSS and non-invasive infections were collected by the Working Group for Beta-hemolytic Streptococci in Japan, as previously reported [30]. *Escherichia coli* DH5α was used as a host for plasmid construction and was grown in a Luria-Bertani liquid medium with shaking or on agar plates at 37°C. *S. pyogenes* was cultured in Todd-Hewitt broth supplemented with 0.5% yeast extract (THY medium) without agitation or on tryptic soy agar supplemented with 5% sheep blood. Cultures were grown at 37°C in a 5% CO_2_ atmosphere. When required, antibiotics were added to the medium at the following final concentrations: erythromycin, 300 µg/mL for *E. coli* and 1 µg/mL for *S. pyogenes*; and spectinomycin (Sp), 25 µg/mL for each of *E. coli* and *S. pyogenes*. The growth of *S. pyogenes* was turbidimetrically monitored at 600 nm, using a MiniPhoto 518R (Taitec, Tokyo, Japan).

### DNA sequencing and data deposit

The nucleotide sequences of the *csrS*, *csrR*, and *rgg* genes were determined by automated sequencers, i.e., an Applied Biosystems 3130xl Genetic Analyzer and an Applied Biosystems 3130 Genetic Analyzer (both Applied Biosystems, Tokyo, Japan). Sequencing data were deposited in the DNA Data Bank of Japan (DDBJ).

### Animals

Male five to six-week-old outbred ddY and hairless mice were purchased from SLC (Shizuoka, Japan) and maintained in specific pathogen-free (SPF) conditions. All animal experiments were performed according to the guidelines of the Ethics Review Committee of Animal Experiments of the National Institute of Infectious Diseases, Japan.

### Construction of deletion or deficient mutants

#### (i) Construction of the *rgg* mutant

A 692-bp DNA fragment containing the internal region of *rgg* was amplified from the NIH34 (for *emm3*) and NIH186 (for *emm1*) chromosomal DNA, using the primers of rgg-del1 and rgg-del2 (Table S3). The PCR products were digested by *Bam*HI and *Eco*RI. This fragment was then cloned into the integration shuttle vector pSF152 [31] to create the plasmid pSF152*rgg3* and pSF152*rgg1*, respectively, which was then used for the chromosomal inactivation of the *rgg* gene, as described previously [31]. The inactivated mutant strains K33*rgg*, OT22*rgg*, S1*rgg*, Se235*rgg* and F482*rgg* (*rgg*::*aad9* Sp^r^) were then selected by using spectinomycin-containing agar plates. Deficiency of the native *rgg* gene was verified by PCR.

#### (ii) Construction of the *csrS* mutant

A 930-bp DNA fragment containing the internal region of *csrS* was amplified from the K33 chromosomal DNA, using the primers of csrS-def1 and csrS-def2 (Table S3). The PCR products were digested by *Bam*HI and *Eco*RI. This fragment was then cloned into the integration shuttle vector pSF152 to create the plasmid pSF152*csrS*, which was then used to create K33*csrS*, as described above.

#### (iii) Construction of the *slo* mutant

A 1,061-bp DNA fragment containing the internal region of *slo* was amplified from the NIH34 chromosomal DNA, using the primers of slo-del3 and slo-del4 (Table S3). The PCR products were digested by *Bam*HI and *Eco*RI. This fragment was then cloned into the integration shuttle vector pSF152 to create the plasmid pSF152*slo*, which was then used to create NIH34*slo*, as described above.

#### (iv) Construction of the *scpC* mutant

A 1,240-bp DNA fragment containing the internal region of *scpC* was amplified from the NIH34 chromosomal DNA, using the primers of scpC-del5 and scpC-del6 (Table S3). The PCR products were digested by *Bam*HI and *Eco*RI. This fragment was then cloned into the integration shuttle vector pSF152 to create the plasmid pSF152*scpC*, which was then used to create NIH34*scpC*, as described above.

#### (v) Construction of the *sdn* mutant

A 693-bp DNA fragment containing the internal region of *sdn* was amplified from the NIH34 chromosomal DNA, using the primers of sdn-def3 and sdn-def2 (Table S3). The PCR products were digested by *Bam*HI and *Eco*RI. This fragment was then cloned into the integration shuttle vector pSF152 to create the plasmid pSF152*sdn*, which was then used to create NIH34*sdn*, as described above.

#### (vi) Construction of the *nga* mutants

A 1,071-bp DNA fragment containing the 5′ terminal of *nga* and the adjacent upstream chromosomal DNA was amplified from the NIH34 chromosomal DNA, using the primers of ngadel1 and ngadel2 (Table S3); additionally, a 775-bp fragment containing the 3′ terminal of *nga* and the adjacent downstream chromosomal DNA was amplified from the NIH34 chromosomal DNA, using the primers of ngadel3 and ngadel4 (Table S3). These two PCR products were digested by *Bam*HI and *Eco*RI and by *Eco*RI and *Pst*I, respectively. The digested fragments were cloned into the erythromycin-resistant and temperature-sensitive shuttle vector pJRS233 [32], to create the plasmid pJRSΔ*nga*. This plasmid was then introduced into the strain NIH34 by electroporation, and transformants were selected on erythromycin agar plates at 30°C. To create an integration of pJRSΔ*nga* with the chromosome, transformants were grown at 39°C and selected with erythromycin. Replacement of the native *nga* gene by the *nga*-deleted mutant allele was verified by PCR, and the resultant strain was named NIH34*nga*.

### Construction of strains integrating the intact *rgg* gene

The replacement of a mutated *rgg* gene by an intact *rgg* gene was performed by allelic recombination. Specifically, the chromosomal DNA derived from the GAS strains K33 (for *emm3*) and F482 (for *emm1*) was purified and used as a template for the PCR amplification of the intact *rgg* gene. The primers used were 5′-GGGGATCCTTATGGCTATATCATAGCTG-3′ (sense) and 5′-GGGAATTCTGTTGAGATAAACTACACC-3′ (antisense). The PCR fragment was ligated into the plasmid pSF152, and the resultant plasmids pSF*rgg3+* (for *emm3*) and pSF*rgg1*+ (for *emm1*) were used for chromosomal integration into the mutated *rgg* gene of isolates from STSS patients, as described previously [31]. The integrated strains (Sp^r^) were then selected by using spectinomycin (Sp)-containing agar plates. Integration of the intact *rgg* gene was confirmed by PCR.

### Western blotting

A total of 1 mL of the supernatant of an overnight bacterial culture (OD_600_ = 1.0) was passed through a 0.45-mm pore size membrane filter (Nippon Millipore, Tokyo, Japan), to remove the remaining cells. Proteins in the resulting cell-free supernatant were precipitated with 10% trichloroacetic acid and resuspended in a sample loading buffer containing a saturated Tris base. Samples were heated at 100°C for 3 min and separated on sodium dodecyl sulfate (SDS)–12.5% polyacrylamide gels. To detect SLO, the proteins on the gels were electrophoretically transferred onto a PVDF membrane. The membrane was blocked with 5% nonfat milk +0.2% Tween-20 and reacted with primary anti-SLO polyclonal antibody (American Research Products, Belmont, MA, USA), secondary antibody peroxidase-conjugated anti-rabbit Ig (GE Healthcare, Tokyo, Japan), and an ECL Plus Western blotting Detection System (GE Healthcare).

### Complete-genome comparisons

Complete-genome comparisons were performed with an array-based service (CGS) provided by NimbleGen Systems Inc. (Madison, WI, USA) [9]. The reference genome sequence used in the microarray was that of *S. pyogenes* SSI-1 (GenBank accession No. BA000034).

### Quantitative RT-PCR analysis

Total RNA was extracted from bacterial cells using the RNeasy Protect Bacteria Mini Kit (QIAGEN, Tokyo, Japan), according to the manufacturer's instructions. Complementary DNA synthesis was performed with the PrimeScript RT reagent kit (Perfect Real Time) (Takara Bio, Otsu, Japan), also following the manufacturer's instructions. Transcript levels were determined using the ABI PRISM Sequence Detection System 7000 (Applied Biosystems) and Premix Ex Taq (Perfect Real Time) (Takara). For real-time amplification, the template was equivalent to 5 ng of total RNA. Measurements were performed in triplicate; a reverse-transcription-negative blank of each sample and a no-template blank served as negative controls. The primers and probes used are listed in Table S4.

### GAS infection in a mouse model

GAS was grown to late-log phase (OD600 = 0.6−0.8) at 37°C in a 5% CO_2_ atmosphere, pelleted by centrifugation, washed twice with sterile phosphate-buffered saline (PBS), suspended in sterile PBS. A total of 1×10^7^ CFU of GAS suspended in 0.5 mL of PBS was injected intraperitoneally into five to six-week-old ddY outbred male mice (10–16 mice/GAS isolate). The number of surviving mice was compared statistically, using the Kaplan-Meier log-rank test. For the subcutaneous infection model, male hairless mice Hos:Hr-1 were injected with 1×10^7^ CFU of GAS in a 100-µl suspension of GAS in PBS. The lesion area was measured daily and analyzed. Dissemination in kidney and spleen of GAS was evaluated by colony counting at day 7 post-infection.

### Histopathological examination

For histopathological analysis, the tissues from GAS-infected mice were directly fixed in 10% neutral-buffered formalin, embedded in paraffin, sectioned and stained with hematoxylin and eosin (H&E).

### Isolation of human neutrophils

Human neutrophils were isolated from the venous blood of five healthy volunteers, in accordance with a protocol approved by the Institutional Review Board for Human Subjects, National Institute of Infectious Diseases [2]. This study complies with the guidelines of the declaration of Helsinki.

### Migration assay

Chemotaxis assays were performed as previously described [2]. Briefly, 5×10^5^ neutrophils in Roswell Park Memorial Institute (RPMI) medium containing 25 mM HEPES and 1% FCS in Transwell inserts (3-µm pore size; Coaster, Corning, NY, USA) were placed in 24-well plates containing 600 µl medium or 100 nM interleukin (IL)-8 solution (Pertec, London, UK); the plates were then incubated with or without 5×10^6^ bacteria for 1 h at 37°C, in advance of the assay. After 1 h of incubation, cells in the lower wells were collected and 10^4^ 10-µm microsphere beads (Polysciences Inc., Warrington, MA, USA) were added. Cells were stained with propidium iodine (Sigma, St Louis, MO, USA) for flow cytometry to quantify the viable neutrophils; analysis was performed, using the FACS Calibur (BD Biosciences, San Jose, CA, USA).

### Accession numbers

The DNA Data Bank of Japan (DDBJ) (http://www.ddbj.nig.ac.jp/index-e.html) accession numbers for the genes and gene products discussed in this paper are: TK283 *csrR* locus - AB517797; TK929 *csrR* locus - AB517804; NIH43 *csrR* locus - AB517807; NIH75 *csrR* locus - AB517814; NIH136 *csrR* locus - AB517819; NIH157 *csrR* locus - AB517822; NIH212 *csrR* locus - AB517826; NIH216 *csrR* locus -AB517827; NIH252-2 *csrR* locus - AB517838; NIH259 *csrR* locus - AB517839; NIH273 *csrR* locus - AB517842; NIH300 *csrR* locus - AB517850; NIH301 *csrR* locus - AB517851; NIH323-1 *csrR* locus - AB517853; NIH381-1 *csrR* locus - AB517863; NIH404 *csrR* locus - AB517867; NIH406 *csrR* locus - AB517868; NIH447 *csrR* locus - AB517877; NIH5 *csrS* locus - AB517796; TK76 *csrS* locus - AB517800; NIH18 *csrS* locus - AB517801; TK280 *csrS* locus - AB517803; NIH35 *csrS* locus - AB517805; NIH44 *csrS* locus - AB517809; NIH49 *csrS* locus - AB517810; NIH55 *csrS* locus - AB517812; NIH75 *csrS* locus - AB517815; NIH102 *csrS* locus - AB517817; NIH152-3 *csrS* locus - AB517820; NIH156-1 *csrS* locus - AB517821; NIH205 *csrS* locus - AB517823; NIH200-4 *csrS* locus - AB517825; NIH220-1 *csrS* locus - AB517828; NIH222 *csrS* locus – AB517829; NIH230 *csrS* locus - AB517830; NIH236 *csrS* locus - AB517831; NIH238 *csrS* locus - AB517833; NIH243 *csrS* locus - AB517834; NIH253-1 *csrS* locus - AB517835; NIH250-2 *csrS* locus - AB517836; NIH263-2 *csrS* locus - AB517840; NIH268 *csrS* locus - AB517841; NIH283-1 *csrS* locus - AB517844; NIH286 *csrS* locus - AB517845; NIH287-1 *csrS* locus - AB517846; NIH296 *csrS* locus - AB517847; NIH297 *csrS* locus - AB517849; NIH317 *csrS* locus - AB517852; NIH325-1 *csrS* locus - AB517854; NIH345 *csrS* locus - AB517855; NIH372 *csrS* locus - AB517859; NIH437 *csrS* locus - AB517862; NIH403 *csrS* locus - AB517866; NIH421 *csrS* locus - AB517871; NIH424-1 *csrS* locus - AB517873; NIH433 *csrS* locus - AB517874; NIH453 *csrS* locus - AB517875; Se202 *csrS* locus - AB517643; NIH3 *rgg* locus - AB517795; NIH8 *rgg* locus - AB517798; TK65 *rgg* locus - AB517799; NIH18 *rgg* locus - AB517802; TK1097 *rgg* locus - AB517806; NIH43 *rgg* locus - AB517808; NIH50 *rgg* locus - AB517811; NIH60 *rgg* locus - AB517813; NIH91 *rgg* locus - AB517816; NIH118 *rgg* locus - AB517818; NIH186 *rgg* locus - AB517824; NIH236 *rgg* locus - AB517832; NIH250.2 *rgg* locus - AB517837; NIH273 *rgg* locus - AB517843; NIH293 *rgg* locus - AB517848; NIH357 *rgg* locus - AB517856; NIH366 *rgg* locus - AB517857; NIH371 *rgg* locus - AB517858; NIH372 *rgg* locus - AB517860; NIH374-2 *rgg* locus - AB517861; NIH381-1 *rgg* locus - AB517864; NIH390 *rgg* locus - AB517865; NIH406 *rgg* locus - AB517869; NIH409 *rgg* locus - AB517870; NIH422 *rgg* locus - AB517872; NIH445 *rgg* locus - AB517876.

## Supporting Information

Table S1 Strains of *emm3* and *emm1* genotype *S. pyogenes* and plasmids used in this study
(0.06 MB DOC)

Table S2 Amino acid difference in comparison with K33
(0.04 MB DOC)

Table S3 Primers used for the construction of deletion mutants
(0.03 MB DOC)

Table S4 Primers used for RT-PCR
(0.06 MB DOC)

Figure S1 Effect of *rgg* mutation of *emm1*-genotyped *S. pyogenes* on survival of human neutrophils. Human neutrophils migrated in the lower wells of a Transwell system in response to IL-8. The migrated human neutrophils were brought into contact with various *emm1* GAS strains (S1, Se235, and F482; non-STSS clinical isolates, NIH60 and NIH186; and STSS isolates and their *rgg* mutants) (Table S1), and then the remaining viable neutrophils were counted. Values shown are means ± SD. *p<0.05, as estimated by Student's *t* test. The results shown are representative of one of four individual experiments, all of which had similar results.
(0.05 MB TIF)
