# Supplementary material for: Highly Frequent Mutations in Negative Regulators of Multiple Virulence Genes in Group A Streptococcal Toxic Shock Syndrome Isolates
Source: PLoS Pathog. 2010 Apr 1;6(4):e1000832. doi: 10.1371/journal.ppat.1000832 (PMC2848555; doi:10.1371/journal.ppat.1000832)
Supplement: Table S1 — Strains of emm3 and emm1 genotype S. pyogenes and plasmids used in this study (0.06 MB DOC) [file ppat.1000832.s001.doc]

Table S1 Strains of *emm3* and *emm1* genotype *S. pyogenes* and plasmids used in this study

| *emm* genotype | Strain Name | Relevant characteristics^a^ | Reference |
| --- | --- | --- | --- |
| *emm3* | C500 | Pharyngitis | This study |
|  | OT22 | Tonsillitis | This study |
|  | K33 | Pharyngitis | [40] |
|  | NIH1 | STSS | [40] |
|  | NIH3 | STSS | [40] |
|  | NIH8 | STSS | [40] |
|  | NIH34 | STSS | [40] |
|  | NIH152-3 | STSS | [41] |
|  | NIH249 | STSS | This study |
|  | NIH327 | STSS | This study |
|  | NIH352 | STSS | This study |
|  | NIH8*rgg*+ | NIH8 carrying intact *rgg*, Sp^r^ | This study |
|  | NIH34*rgg*+ | NIH34 carrying intact *rgg*, Sp^r^ | This study |
|  | NIH34*csrS* | NIH34 carrying *csrS* deficient mutation, Sp^r^ | This study |
|  | NIH34*slo* | NIH34 carrying *slo* deficient mutation, Sp^r^ | This study |
|  | NIH34*nga* | NIH34 carrying *nga* deletion mutation | This study |
|  | NIH34*scpC* | NIH34 carrying *scpC* deficient mutation, Sp^r^ | This study |
|  | OT22*rgg* | OT22 carrying *rgg* deficient mutation, Sp^r^ | This study |
|  | K33*rgg* | K33 carrying *rgg* deficient mutation, Sp^r^ | This study |
|  | Plasmid name | Relevant characteristics | Reference |
|  | **pSF152** | integration shuttle vectors | [31] |
|  | pJRS233 | temperature-sensitive shuttle vector | [32] |
| *emm1* | S1 | Pharyngitis | This study |
|  | Se235 | Pharyngitis | This study |
|  | F482 | Pharyngitis | This study |
|  | NIH60 | STSS | This study |
|  | NIH186 | STSS | [34] |
|  | S1*rgg* | S1 carrying *rgg* deficient mutation, Sp^r^ | This study |
|  | Se235*rgg* | Se235 carrying *rgg* deficient mutation, Sp^r^ | This study |
|  | F482*rgg* | F482 carrying *rgg* deficient mutation, Sp^r^ | This study |
|  | NIH60*rgg*+ | NIH60 carrying intact *rgg*, Sp^r^ | This study |
|  | NIH186*rgg+* | NIH186 carrying intact *rgg*, Sp^r^ | This study |

a: Strains were isolated from symptomatic patients with STSS or non-STSS (Table 1).

Sp^r^; spectinomycin resistance. STSS, streptococcal toxic shock-like syndrome [8]
