# Supplementary material for: Highly Frequent Mutations in Negative Regulators of Multiple Virulence Genes in Group A Streptococcal Toxic Shock Syndrome Isolates
Source: PLoS Pathog. 2010 Apr 1;6(4):e1000832. doi: 10.1371/journal.ppat.1000832 (PMC2848555; doi:10.1371/journal.ppat.1000832)
Supplement: Table S2 — Amino acid difference in comparison with K33 (0.04 MB DOC) [file ppat.1000832.s002.doc]

Table S2 Amino acid difference in comparison with K33

| Genome position^a^ | DNA change | Gene | Change of NIH1 | NIH1 ^b, c^ | K33 ^b, c^ | C500 ^b, c^ |
| --- | --- | --- | --- | --- | --- | --- |
| 3676 | C→T | GTP-binding protein (*SPs0004*) | Nonsynonymous | Y | N | Y |
| 137628 | A→C | V-type ATP synthase subunit B (*SPs0123*) | Nonsynonymous | Y | N | Y |
| 137792 | A→G | V-type ATP synthase subunit B (*SPs0123*) | Synonymous | N | N | N |
| 362212 | A→G | *codY* (*SPs0322*) | Nonsynonymous | Y | N | N |
| 396071 | A→C | *SPs0358* | Synonymous | N | N | N |
| 687764 | G→A | peroxide resistance protein (*SPs0680*) | Nonsynonymous | Y | N | Y |
| 763994 | G→A | transcriptional regulator (*SPs0776*) | Synonymous | N | N | N |
| 786071 | A→G | tRNA ribosyltransferase-isomerase (*SPs0797*) | Synonymous | N | N | N |
| 1612763 | A→G | *csrR* (*SPs1615*) | Nonsynonymous | Y | N | N |
| 1757412 | G→A | *rgg* (*SPs1742*) | Nonsynonymous | Y | N | N |

^a^ Location in NIH1 (SSI-1) genome

^b^ Y, there is an amino acid substitution within the ORF of NIH1 or C500 in comparison with K33. N, there is not an amino acid substitution within the ORF in comparison with K33

^c^ NIH1 is an STSS isolates, while K33 and C500 are non-STSS isolates.
