# Supplementary material for: Highly Frequent Mutations in Negative Regulators of Multiple Virulence Genes in Group A Streptococcal Toxic Shock Syndrome Isolates
Source: PLoS Pathog. 2010 Apr 1;6(4):e1000832. doi: 10.1371/journal.ppat.1000832 (PMC2848555; doi:10.1371/journal.ppat.1000832)
Supplement: Table S3 — Primers used for the construction of deletion mutants (0.03 MB DOC) [file ppat.1000832.s003.doc]

Table S3 Primers used for the construction of deletion mutants

| Primer | Sequence (5′–3′) |
| --- | --- |
| rggdel1 | GGGGATCCTAGATTAAACAAGTTTGTGGTG |
| rggdel2 | GGGAATTCTGCTCAGCCCCCTATTTACC |
| csrSdef1 | GGGGATCCTAAGAGAACTTAGCAGAAG |
| csrSdef2 | GGGAATTCTTAAATACGGGCTATCGTTTTTGG |
| slo-del3 | GGGGATCCCTCCCAAAGAAATGCCAC |
| slo-del4 | GGGAATTCTGAAATAGGATAAGCTGGG |
| scpC-del5 | GGGGATCCGTGGCTTTAGGAGCAGATGTG |
| scpC-del6 | GGGAATTCGAGAAGTCAAGGTGAAGCGG |
| sdn-def1 | GGGGATCCCATTGACTAATCTGCTTTTCC |
| sdn-def2 | GGGAATTCCTTTCTGTTAAGAGTCCGCCC |
| nga-del1 | GGGGATCCGTCCAGAGTCGTCACGAACC |
| nga-del2 | GGGAATTCCATGTAAACCACCTTATATTA |
| nga-del3 | GGGAATTCTAACAATATGTATAAGGTGCC |
| nga-del4 | GGCTGCAGATTCTAGTGGCATTTCTTTGGG |
