# Supplementary material for: Highly Frequent Mutations in Negative Regulators of Multiple Virulence Genes in Group A Streptococcal Toxic Shock Syndrome Isolates
Source: PLoS Pathog. 2010 Apr 1;6(4):e1000832. doi: 10.1371/journal.ppat.1000832 (PMC2848555; doi:10.1371/journal.ppat.1000832)
Supplement: Table S4 — Primers used for RT-PCR (0.06 MB DOC) [file ppat.1000832.s004.doc]

Table S4 Primers used for RT-PCR

| gene | Primer | Sequence (5′–3′) |
| --- | --- | --- |
| *grab* | grab-RTP1 | GCTGTTGACTCACCTATCGAACA |
|  | grab-RTP2 | CTGGAGCATTGCCAAGAAGAT |
| *gyrA* | gyrA-RTP1 | CATGAGTGTCATTGTGGCAAGA |
|  | gyrA-RTP2 | CGACGATGCACAGGTTTCAG |
| *mac* | mac-RTP1 | CTTCCGTTTGGACCAAAGGA |
|  | mac-RTP2 | TAAGGAGCGCGAAAAACATCTT |
| *nga* | nga-RTP1 | ACGTACGCTGTTAGTGGCAAAG |
|  | nga-RTP2 | AGGAAGTTGCGTTAGCTTCCAT |
| *sagA* | sagA-RTP1 | CTGCTGTTGCTGCTGTACTACTTG |
|  | sagA-RTP2 | TTCCGCTACCACCTTGAGAATT |
| *scpA* | scpA-RTP1 | GACAGCTGACGGCAATATTAAGC |
|  | scpA-RTP2 | TGTTAGCCACTGACGACAAAATATC |
| *scpC* | spyCEP-RTP1 | AGGAGCTTGGGACAAGGGATA |
|  | spyCEP-RTP2 | TGATGGGCCGGATCGAT |
| *sdn* | sdn-RTP1 | GCTTAGAAACTCTCTCGCCAGAA |
|  | sdn-RTP2 | GGAATCGCCATCACAGCAA |
| *ska* | ska-RTP1 | GCTGACAAAGATGGTTCGGTAAC |
|  | ska-RTP2 | CATGCCCGCTTAGCAAAAAT |
| *slo* | slo-RTP1 | AAAACAAACCAGACGCGGTAGT |
|  | slo-RTP2 | TGCTTTGTCTCCCATACCTGGTA |
| *speA* | speA-RTP1 | TTCTTTATGAGGGTGACCCTGTTAC |
|  | speA-RTP2 | CCCTGAAACATTATATATTAAATCGTGAGA |
| *speB* | speB-RTP1 | CGCACTAAACCCTTCAGCTCTT |
|  | speB-RTP2 | ACAGCACTTTGGTAACCGTTGA |
| TaqMan probe |  |  |
| *grab* | grab-TMP1 | CCTCGAATTATTCCAAATGGCAGAACCTTAACT |
| *gyrA* | gyrA-TMP1 | CTTTGCCAGATGTGCGTGATG |
| *mac* | mac-TMP1 | TTACTCCTCCAGCAAAATTCACTCAA |
| *nga* | nga-TMP1 | TAAAAAAAGCGATGTCAAATATGAAACGACCAAAGT |
| *sagA* | sagA-TMP1 | TGCTTCTCAATTGCTACTGGAAGTG |
| *scpA* | scpA-TMP1 | AGATATTGCAGCACCCGGCCA |
| *scpC* | spyCEP-TMP1 | AAGGACAAGGCAAGGTTGTCGCAGTTATTG |
| *sdn* | sdn-TMP1 | AGTTGTTGCAGCATTCGCATCA |
| *ska* | ska-TMP1 | TGCCGACCCAACCTGTCCAAG |
| *slo* | slo-TMP1 | ACCAAGCGAAACCCACAAAAAATCCATATTG |
| *speA* | speA-TMP1 | CACGAGAATGTGAAATCTGTTGATCA |
| *speB* | speB-TMP1 | TACTGGTGGCGGCGCAGG |
