# Supplementary figures and images for: Highly Frequent Mutations in Negative Regulators of Multiple Virulence Genes in Group A Streptococcal Toxic Shock Syndrome Isolates
Source: PLoS Pathog. 2010 Apr 1;6(4):e1000832. doi: 10.1371/journal.ppat.1000832 (PMC2848555; doi:10.1371/journal.ppat.1000832)

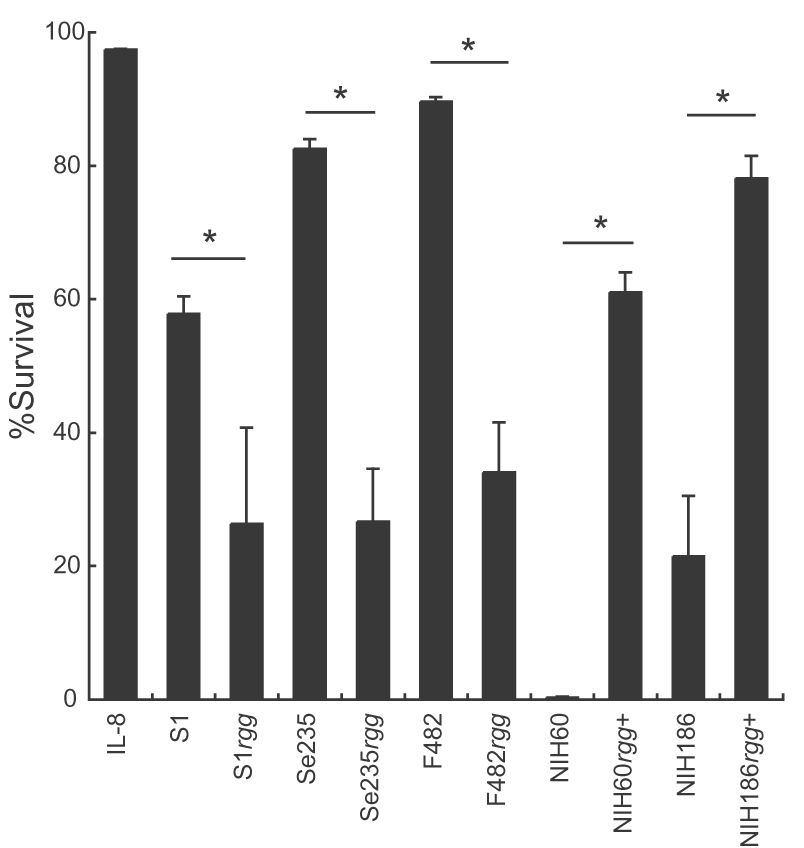

Supplement: Figure S1 — Effect of rgg mutation of emm1-genotyped S. pyogenes on survival of human neutrophils. Human neutrophils migrated in the lower wells of a Transwell system in response to IL-8. The migrated human neutrophils were brought into contact with various emm1 GAS strains (S1, Se235, and F482; non-STSS clinical isolates, NIH60 and NIH186; and STSS isolates and their rgg mutants) (Table S1), and then the remaining viable neutrophils were counted. Values shown are means ± SD. *p<0.05, as estimated by Student's t test. The results shown are representative of one of four individual experiments, all of which had similar results. (0.05 MB TIF) [file ppat.1000832.s005.tif]
